# Neuroaesthetics and the Trouble with Beauty

**DOI:** 10.1371/journal.pbio.1001504

**Published:** 2013-03-19

**Authors:** Bevil R. Conway, Alexander Rehding

**Affiliations:** 1 Program in Neuroscience, Wellesley College, Wellesley, Massachusetts, United States of America; 2 Department of Neurobiology, Harvard Medical School, Boston, Massachusetts, United States of America; 3 Department of Music, Harvard University, Cambridge, Massachusetts, United States of America

## Abstract

Neuroscience is increasingly being called upon to address issues within the humanities. We discuss challenges that arise, relating to art and beauty, and provide ideas for a way forward.

The famous nineteenth-century psychophysicist Gustav Fechner was also a poet and art critic. Armed with the tools of science, Fechner sought to reconcile his various interests. He would doubtless be interested by technological developments in neuroscience that have revealed the operations of neurons at cellular resolution and have enabled us to peer almost unnoticed into each other's working brains. But can these tools advance our understanding of aesthetics beyond Fechner's insights [1]? The nascent field of neuroaesthetics claims it can. Here we consider what questions this new field is poised to answer. We underscore the importance of distinguishing between beauty, art, and perception—terms often conflated by “aesthetics”—and identify adjacent fields of neuroscience such as sensation, perception, attention, reward, learning, memory, emotions, and decision making, where discoveries will likely be informative.

## Aesthetics and Neuroscience

Aesthetics has a complex history. The term derives from the Greek “perception” and was coined by Alexander Baumgarten in 1750 as the study of sensory knowledge. But following Immanuel Kant's *Critique of Judgment* in 1790 [2], aesthetics began focusing on the concept of beauty, in nature and in art. During the nineteenth century, the term became largely synonymous with the philosophy of art. These three connotations—perception, beauty, art—point in different directions but are often conflated in neuroaesthetics.

Kant is a preferred philosopher among neuroaestheticians, no doubt because of his towering stature in the history of Western thought. He pursued a universalist approach to beauty, an appealing concept for neuroscientists because it suggests a discrete neural basis. But Kant's concept of beauty has been severely criticized in light of the prevailing pluralism of artistic styles. To make matters more complicated, there is no consensus on the nature of beauty. Kant's understanding of beauty was predicated on an attitude of “disinterested contemplation” [2], whereas Friedrich Nietzsche roundly dismissed this notion and underlined the impact of sensual attraction [3]. For the poet John Keats, beauty equaled truth [4], while Stendhal, the French novelist, characterized beauty as the “promise of happiness” [5]. More recently, Elaine Scarry described beauty as an urge to repeat [6]. While each of these theories is respected, not one is universally accepted. Partly this diversity of opinions is connected to the different functions that beauty holds within various philosophical systems, being sometimes viewed in connection with epistemology or with ethics. One goal of neuroaesthetics is to get to the bottom of the problem of artistic beauty. How can this be accomplished?

Experiences of beauty are often deeply moving, and their importance to the human condition invites a neuroscientific explanation. But while deep emotional reactions are often associated with beauty, being moved does not always indicate an instance of beauty. Consider hearing about a disaster, celebrating a sports victory, or smelling a long-forgotten scent. These experiences are better described as “sympathy,” “elation,” and “memory,” rather than experiences of beauty. If neuroaesthetics is to be concerned specifically with beauty, it must draw distinctions between mechanisms for such disparate reactions. Since many experiences of beauty are related to art, neuroaestheticians have focused their attention on the analysis of artworks. For example, Ramachanran [7], Zeki [8], and Kandel [9] have presented case studies focusing on classical Indian art, American and European modernists, and the Viennese Secessionists. Explicitly or implicitly, these studies aim to extract rules that would lead to a practical definition of beauty, connecting features of objects and neural activity. Zeki, for instance, argues that the power of Alexander Calder's sculptures derives from the black-and-white moving parts, potent activators of the brain's motion-processing center.

**Figure 1:**
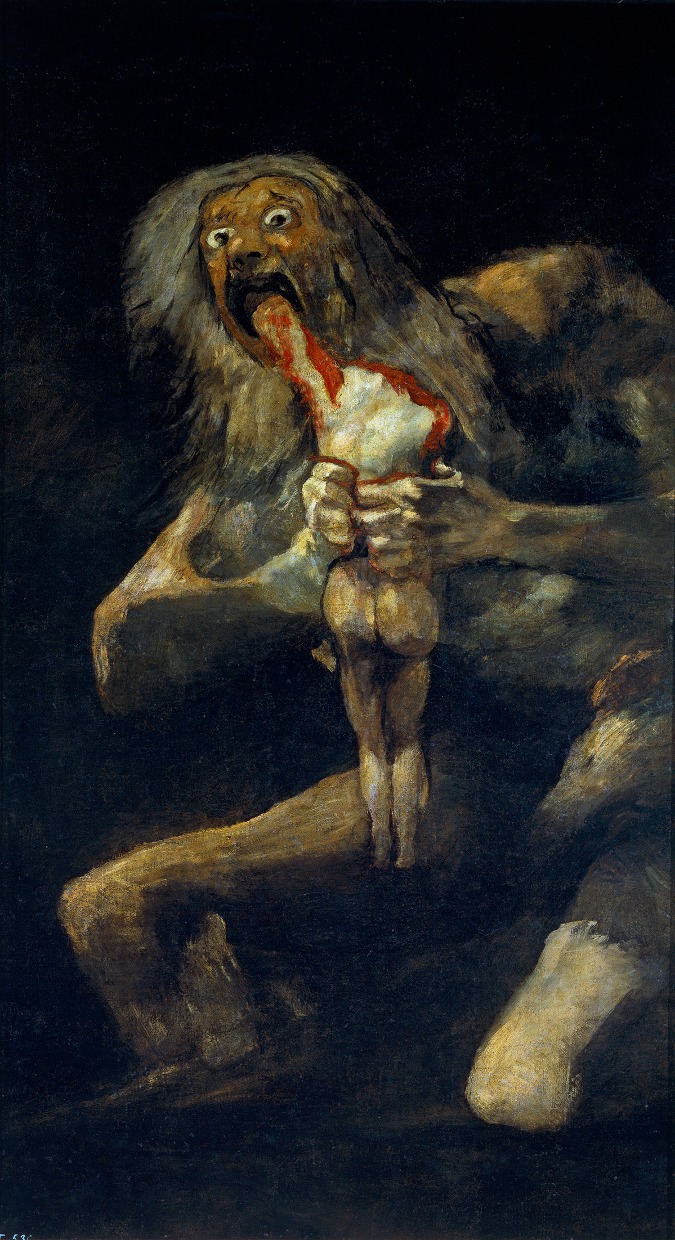
Goya y Lucientes, Francisco de, *Saturn devouring one of his sons* (1821–1823). Mural transferred to canvas. 143.5 cm×81.4 cm. Museo del Prado, Madrid.

It may be no coincidence that the art these three authors hold up relates to the culture in which they were each raised. One potential danger in aesthetic projects is to universalize one's subjective convictions and assume that an experience of beauty is common to all. Projecting from individual subjective experience is deceptive, for there is ample evidence that notions of beauty vary between cultures and are mutable even within a culture—just think of fast-changing trends in fashion. Moreover, the equation (art = beauty) rests on shaky ground. Throughout history, artists have created deeply moving artwork that is emphatically not beautiful; Goya's *Saturn Devouring One of His Sons* (Figure 1) provides a famous historical example. Large swaths of twentieth-century art have greatly expanded—or entirely disavowed—notions of beauty. Such distinctions may seem picky, but interdisciplinary work such as neuroaesthetics relies on shared principles, and requires heightened attention to conceptual clarity.

Neuroscience has provided a heuristic outlining how sensory signals are processed by the nervous system to yield behavior [10],[11]. Signals from sensory epithelia such as the retina or basilar membrane are processed in the cerebral cortex by a series of areas that compute descriptions of the world: what or where objects are. These brain areas send signals to other brain structures that are responsible for evaluating options against expected rewards—attaching significance to the sensory descriptions—and ultimately for making decisions, guided by learning, memory, and emotions. Below we argue that a successful neuroaesthetics will include the study of each of these stages of processing as they relate to handling, encoding, and generating aesthetic experiences, rather than an attempt to derive a single universal neural underpinning of what constitutes beauty.

## First Steps in Neuroaesthetics: Sensation, Perception, and Art

One approach commonly included under the umbrella of neuroaesthetics involves examining art objects in museums. Here the complication of establishing “beauty” is obviated by treating artworks as products of a massive empirical experiment. By analogy with evolutionary theory, the assumption is that the tiny number of works that survive the selective pressures exerted by collectors, cultural institutions, and fads are enriched for the strength of their effects on the nervous system. Using this approach, studies have uncovered various artistic strategies reflecting fundamental operations of the neural mechanisms for sensation and perception [7],[8],[12]–[14]. For example, depictions of shadows in paintings often do not correspond to the light sources that cause them [15]. Such unnoticed deviations from veracity reveal important adaptations of the brain to ecological pressures during evolution and development—in the case of shadows, the relationship of objects to light sources is in flux and therefore not a stable feature. Similarly, analysis of portraits has been insightful, showing that the outer contour of a face is more important for face recognition than the precise configuration of features [16]. And paintings by Paul Cezanne, Henri Matisse, and Claude Monet show how these artists capitalized upon the neural mechanisms of color [17]. This line of research is often described as the neuroscience of art, rather than neuroaesthetics, since it does not test for beauty [13]. The approach may reveal the perceptually relevant properties of visual stimuli—contributing to aesthetics as Baumgarten defined it—but these properties are neither necessary nor sufficient features of beautiful objects. An Alexander Calder sculpture may consist of optimal stimuli for the brain's motion center, but this aspect of the work does not make it beautiful. The art simply provides a fascinating demonstration of the computations of the brain's motion-perception circuits, and the genius of the artists for discovering them.

It is an open question whether an analysis of artworks, no matter how celebrated, will yield universal principles of beauty. Compositional principles such as the golden ratio are intriguing possible universals, and captured the attention of Fechner, but despite mathematical appeal, the golden rectangle is not the favorite rectangle shape of most people [18]. One possible almost-universal may be the appeal of certain female facial features (symmetry, high cheekbones, large eyes) and a 0.7 waist-to-hip ratio [19] or high body mass index [20]. Explanations for these preferences depend on a correlation between the attributes and reproductive fitness. Yet celebrated representations of female beauty across history can deviate considerably from the 0.7 rule, and ratio preferences vary across cultures [21],[22]. Depictions of reproductive fitness can be sexually appealing and contribute to aesthetic appeal, but such depictions are, again, neither necessary nor sufficient for beauty. Another possible universal concerns the intriguing discovery that painters typically center one eye along the horizontal axis of a picture [23], taken to indicate “hidden principles…operating in our aesthetic judgments.” But the trend towards eye-centering has declined dramatically during and after avant-garde movements such as those led by Picasso [13]. Whether this decline is attributable to the relative decline of beauty as a driving force in artistic creation or indicates a cultural shift in aesthetic preferences is unclear. Using celebrated works as empirical data to understand beauty might be a worthwhile gambit, but we doubt that conclusions can be extended across peoples, times, and cultures. The only universal feature of beauty besides our capacity to experience it appears to be its mutability, itself perhaps a topic for neuroscience.

## A Beauty Center?

Fechner was well aware of the pitfalls of philosophical aesthetics and aimed to reformulate the field “from the ground up.” His appreciation of the inherently subjective nature of beauty led him to start with feelings of pleasure and displeasure elicited by art, since these constituted for him the bottom line beyond which further analysis was impossible. Contemporary neuroscience has gone much further. A recent study claims that “all works that appear beautiful to a subject have a single brain-based characteristic, which is that they have as a correlate of experiencing them a change in strength of [fMRI] activity within the mOFC [medial orbitofrontal cortex]” [24]. Leaving aside methodological challenges [25],[26], is such a correlation meaningful to understanding aesthetics?

Subjectivist studies such as these overcome the difficulty of defining beauty by asking the participants to first rate visual objects or sounds [24],[27]. Brain activity of each subject is then assessed to their own set of “beautiful” versus “ugly” stimuli. Four experimental-design challenges surface. First, the options are necessarily restricted, and might not include a truly beautiful choice—the study design tests preferences, not beauty. Second, different subjects likely interpret the instructions in radically different ways. Third, the use of different stimulus sets in different subjects makes it difficult to control for differences in low-level stimulus features, which likely drive different patterns of neural activity. And fourth, the experiment requires that a given object retain a fixed preferred status, and one that is not modulated by context, which we know is unlikely. As Fechner showed, mere exposure changes judgments of preference in favor of the familiar option. Brandishing fMRI does not circumvent these problems. Moreover, fMRI has cripplingly low spatial and temporal resolution, and the relationship between the measured signal and underlying neural activity is indirect. In addition, fMRI experiments often only report regions that show differential activation between pairs of conditions (e.g., response to beautiful greater than response to ugly); such an analysis is misleading in situations in which all brain regions show significant but slightly different levels of activity for the different conditions, as is likely the case in considerations of beauty. Brain imaging provides a blurry, although seductively glossy, view of brain function. And by finessing a definition of beauty, these sorts of studies sidestep what is at the heart of our interest in beauty: the connection between physical stimuli, specifically those crafted by human hands, and our response.

Nonetheless, a discovery that every person's experience of beauty (however vaguely defined) correlates with activity within a specific brain region would be surprising, since it would seem more likely that a complex reaction (beautiful!) would hinge not on the absolute level of activity within a single brain center but rather on the pattern of activity across many distributed brain regions—specifically those responsible for perception, reward, decision making, and emotion. Indeed, a broader reading of the literature reveals that the mOFC is not uniquely associated with experiences of beauty and may be neither necessary nor sufficient for these experiences. The mOFC appears to be part of a large network of brain regions that subserves all value judgments. For example, elevated activity within the mOFC is reported in studies of neuroeconomics in which subjects are asked to assign value to a selection of choices and are never asked to consider the beauty of the choices [11],[28]–[30]. The mOFC has also been implicated in impulse control and self-regulation [31], in changing decision thresholds that influence whether information should be expressed in an evaluation [32], in attentional processes that underlie emotion-congruent judgment [33], and in moral decision making [34]. Ascribing responses of the mOFC to experiences of beauty is premature; many experiences depend on these processes without being beautiful [27],[35]–[38].

If the mOFC plays a critical role in mediating beauty, one might expect that strokes of the region would impair experiences of beauty. Strokes of the mOFC are rare, but the limited evidence suggests they affect self-related systems such as self-evaluation [39],[40] and do not impact a person's ability to experience beauty. Alternatively, strokes in other brain regions can, paradoxically, enhance creativity, providing support for the notion that the expression of beauty depends on a broad, distributed network. Frontotemporal dementia can produce an acquired obsessiveness that is often linked to enhanced art production, usually of extremely detailed works [41]. In addition, strokes of the left hemisphere, which often cause aphasia, can produce hyperexpressiveness [42].

## What Questions Can Neuroaesthetics Answer?

Inspired by the power of polling, in 1994 a pair of artists, Komar and Melamid, set out to determine “USA's most wanted painting.” The painting was formulated on the basis of a thousand people's responses to questions of their favorite color, favorite setting, and favorite subjects. The resulting painting is absurd, showing that a composition with everything that people find beautiful does not make a beautiful painting. Rational reductionist approaches to the neural basis for beauty run a similar risk of pushing the round block of beauty into the square hole of science and may well distill out the very thing one wants to understand. There is a popular conception of beauty as a fixed attribute of objects, a notion that much of current neuroaesthetics depends upon. But there is a distinction between abstract notions of beauty and our experience of it—consider a specific example in which you have experienced beauty. Beauty is an analog, not binary, condition that varies in complex ways with exposure, context, attention, and rest—as do most perceptual responses. In trying to crack the subjective beauty nut with scientific, objective information, we also run the risk of fueling a normative, possibly dangerous campaign through which science is required to valorize our experience. Should we deny someone's experience of beauty if the mOFC is not activated? Obviously not. But the question underscores the danger of reverse inference, a technique used in brain-imaging studies which posits that activation of a brain region indicates the presence of a stimulus [43]. Reverse inference is almost always invalid because single brain structures almost never regulate single specific experiences.

Insofar as beauty is a product of the brain, correlations between brain activity and experiences of beauty must exist. At what spatial scale, and within what brain regions, do we find these correlations? What functions do the brain regions implicated serve in other behaviors? What signals during development and experience are responsible for wiring up these circuits? And perhaps most critically, how does the activity of these circuits integrate across modalities and time to bring about the dynamic, elusive quality of beauty? To address these questions, the field is thirsty for carefully conducted experiments that distinguish responses to beauty from those involved in more general value-based decision tasks such as self-evaluation or selecting a juice for lunch. But any such experiments are caught on the same stubborn thorn—the lack of a cogent, universally accepted definition of beauty. One should not always demand a precise definition to make headway, but it might turn out that the philosophers' disagreement is symptomatic: maybe there is no universal concept beyond the human capacity to experience beauty. Our caution about neuroscience's focus on beauty differs from the skepticism that attended scientific study of other subjective phenomena such as illusory contours (or even consciousness); in the case of illusory contours, the subjective experience to a given physical stimulus is universal. So, what is neuroaesthetics supposed to study?

Experiences of beauty typically require attention and are accompanied by feelings of pleasure [11],[27],[44]. In the same way that basic studies at the interface of sensory neuroscience and art have been productive—not in addressing why art objects are beautiful but in uncovering the strategies that artists use to generate artwork—basic investigations of the mechanisms of attention, decision making, reward, and emotion [11],[28],[29],[45]–[47] could inform neuroaesthetics. The field will benefit from developing models relating observations from the humanities to the careful neuroscience that has uncovered computations at cellular resolution within the value-judging structures of the monkey brain. These structures, not coincidentally, are analogous to those identified in fMRI studies of beauty in humans. Some neurons within these structures encode the value of the choices on offer, while others encode the value of the selected choice. Moreover, the neurons adapt on different timescales, displaying “menu-invariant” firing at short timescales and adaptable behavior on longer timescales. This adaptation may account for our ability to make choices across vastly different scales, for example from a restaurant menu in one instance and from houses offered for sale in the next instance [48]. It seems entirely reasonable—even likely—that these neurons are also implicated in the thorny task of deciding what is beautiful. Reformulated in this way, neuroaesthetics is decoupled from beauty and can exploit advances across a range of empirical neuroscience, from sensory encoding to decision making and reward.

There may well be a “beauty instinct” implemented by dedicated neural machinery capable of producing a diversity of beauty reactions, much as there is language circuitry that can support a multitude of languages (and other operations). A need to experience beauty may be universal, but the manifestation of what constitutes beauty certainly is not. On the one hand, a neuroaesthetics that extrapolates from an analysis of a few great works, or one that generalizes from a single specific instance of beauty, runs the risk of missing the mark. On the other, a neuroaesthetics comprising entirely subjectivist accounts may lose sight of what is specific to encounters with art. Neuroaesthetics has a great deal to offer the scientific community and general public. Its progress in uncovering a beauty instinct, if it exists, may be accelerated if the field were to abandon a pursuit of beauty per se and focus instead on uncovering the relevant mechanisms of decision making and reward and the basis for subjective preferences, much as Fechner counseled. This would mark a return to a pursuit of the mechanisms underlying sensory knowledge: the original conception of aesthetics.
